# The role of macrophages in vascular calcification: strategies for diagnosis and treatment

**DOI:** 10.3389/fimmu.2025.1724464

**Published:** 2025-12-30

**Authors:** Yingkun Sheng, Yue Qiu, Xiao Wang, Jingyi Shi, Ziyan Yin, Zile Zhang, Shipeng Jiang, Jian Zhang, Xiaoxiao Hu, Weiling Hong

**Affiliations:** 1 Xingzhi College, Zhejiang Normal University, Jinhua, China; 2 Jinhua Advanced Research Institute, Jinhua, China; 3 Jinhua Key Laboratory of Quality Evaluation and Standard Research of Traditional Chinese Medicine, Jinhua Food and Drug Inspection Research Institute, Jinhua, China; 4 School of Engineering, Westlake University, Hangzhou, China

**Keywords:** diagnosis, immunomodulation, macrophage polarization, treatment, vascular calcification

## Abstract

Vascular calcification (VC) is an actively regulated pathological process that significantly increases the risk of cardiovascular events. As key cells of the innate immune system, macrophages play a dual role in VC through polarization into different phenotypes: Pro-inflammatory macrophages promote calcification by secreting pro-inflammatory factors, releasing apoptotic bodies, and producing extracellular vesicles (EVs); conversely, Anti-inflammatory macrophages inhibit calcification through anti-inflammatory factors, exosomes, plaque stabilization, and ATP/pyrophosphate (PPi) metabolism. However, under metabolic diseases such as diabetes, anti-inflammatory macrophages may exhibit pro-calcific properties. This review systematically summarizes the mechanisms of macrophage polarization in VC, discusses the application of macrophage-related biomarkers and imaging techniques in diagnosis, and highlights therapeutic strategies targeting macrophage polarization, recruitment, and activation. Finally, current challenges in dynamically monitoring macrophage polarization and context-dependent functional heterogeneity are outlined, and future research directions focusing on immunomodulation-based multi-target drug design and engineered cell therapies are proposed.

## 1 Introduction

Vascular Calcification (VC) is a common pathological phenomenon, particularly prevalent in patients with atherosclerosis, diabetes, and chronic kidney disease. It significantly elevates the risk of cardiovascular events and mortality (1, 2). Traditionally, VC was regarded as a passive, degenerative process of mineral deposition. However, recent studies have revealed that it is a highly active, cell-driven biological process, resembling bone development and remodeling. This process involves interactions among various cell types and complex regulatory signaling pathways (3, 4).

As a key component of the innate immune system, macrophages play a central role in inflammatory responses, tissue repair, and homeostasis maintenance (5). Their high plasticity enables them to polarize into distinct functional phenotypes, primarily the pro-inflammatory type and the anti-inflammatory/repair-oriented type, in response to microenvironmental signals (6). Two extreme phenotypes have been described *in vitro*, namely pro-inflammatory “M1-like” macrophages and IL-4/IL-13 alternatively activated “M2-like” macrophages (7). Pro-inflammatory macrophages promote calcification through various mechanisms, including the release of pro-inflammatory factors, apoptotic bodies, and EVs (8, 9). In contrast, M2-like macrophages inhibit or delay the progression of calcification via the secretion of anti-inflammatory factors, plaque stabilization, and ATP/PPi metabolism (10–12). However, the functions of macrophages exhibit significant heterogeneity and dual roles across different pathological contexts. Particularly in metabolic diseases such as diabetes, Anti-inflammatory macrophages may even contribute to calcification (13).

Therefore, gaining an in-depth understanding of the specific mechanisms by which macrophages contribute to VC will not only help elucidate the pathological nature of the disease but also provide important directions for developing novel diagnostic markers and therapeutic strategies. This review systematically summarizes the role of macrophage polarization in the mechanisms underlying VC and discusses recent advances and persisting challenges in macrophage-based diagnostic and therapeutic approaches.

## 2 Basic biology of vascular calcification

VC represents an ongoing process whereby hydroxyapatite accumulates as a direct result of altered signaling pathways amidst the intricate interactions between intracellular and extracellular elements. Cells residing in various layers of the vessel wall, along with circulating stem/progenitor cells, serve as a crucial origin of osteoblast-like cells that actively participate in the calcification process (14).

### 2.1 Cellular composition of blood vessels

**Figure 1:**
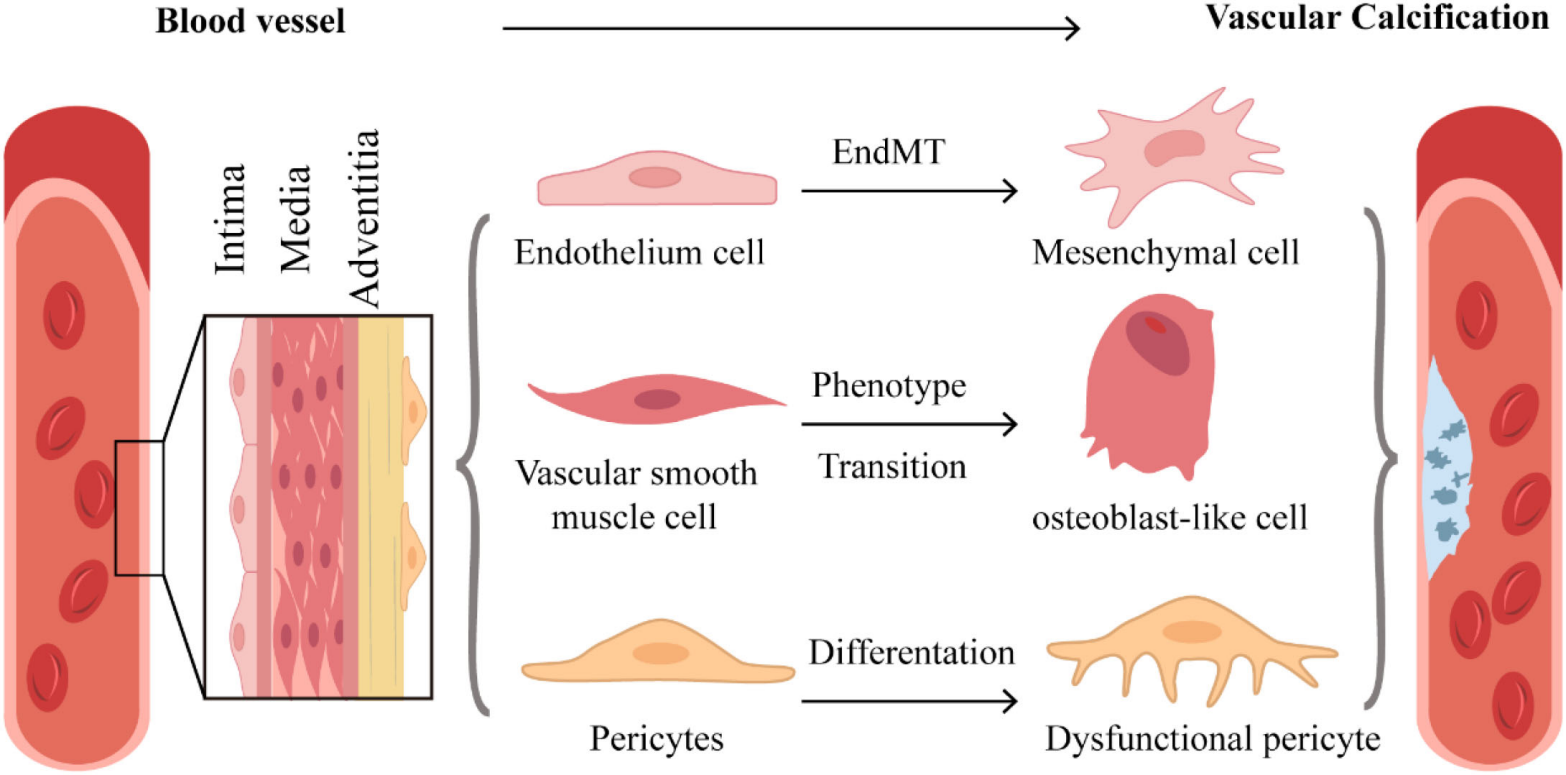
Cellular composition of blood vessels. Blood vessels are composed of cells such as ECs, VSMCs, and PCs. ECs can undergo endothelial-mesenchymal transition (EndMT), while PCs and VSMCs can transform into osteoclast-like cells. These processes are associated with VC.

The inner surface of vessels is lined by a single layer of specialized endothelial cells (ECs), known as the vascular endothelium, which plays crucial roles in various physiological processes, including the regulation of coagulation, maintenance of blood pressure, and stimulation of angiogenesis (15). ECs exhibit significant phenotypic flexibility and are capable of undergoing endothelial-to-mesenchymal transitions (EndMT) (16). EndMT is essential for normal embryogenesis and wound healing, but prolonged or extensive EndMT may lead to the occurrence of pathological events such as VC (17). EndMT involves the activation of kallikreins and elastases, which are proteases mediated by Sox2, and these proteases are capable of inducing VC (18) (**Figure 1**). In addition, EndMT is also a crucial factor affecting mineral deposition, which in turn influences plaque stability (19, 20).Therefore, ECs also play a crucial role in the pathogenesis of VC.

VSMCs are the most commonly used cell type *in vitro* models for studying the mechanisms of VC. VSMCs and extracellular matrix (ECM) compose the medial layer of the vessel wall. VSMCs typically exhibit a contractile phenotype characterized by the expression of a series of cytoskeletal proteins, such as smooth muscle-22α (SM-22α), α-smooth muscle actin (α-SMA), smooth muscle myosin heavy chains (SMHC), calponin (CNN), and smoothelin (SMTN). However, in response to injurious stimuli, oxidative stress, mechanical forces, humoral factors, vasoactive agents, changes in pyrophosphate levels, and inflammatory mediators, VSMCs can differentiate from a contractile phenotype to an osteogenic phenotype (21) (**Figure 1**). The characteristics of the osteogenic phenotypic switch include: (i) decreased expression of contractile proteins and upregulation of mineralization-related markers [Runt-related transcription factor 2 (RUNX2), Osterix (OSX), osteopontin (OPN), osteocalcin (OC), alkaline phosphatase (ALP), SOX9, type II collagen, and type X collagen]; (ii) loss of natural calcification inhibitors such as MGP; (iii) increased matrix metalloproteinases(MMPs); and (iv) secretion of EVs that induce local inflammation and promote VC (22–24).

Pericytes (PCs) are present within the basement membranes of capillaries, small arteries, and post-capillary venules, and are also distributed in the outer layer of the media and the adventitia of large vessels (25).They are considered multipotent cells because they are capable of differentiating into various mature mesoderm-like cell lineages, such as osteoblasts, chondrocytes, smooth muscle cells, adipocytes, and others (26–29). In terms of function, PCs are capable of stabilizing the vascular structure, modulating blood flow within the microcirculation, and regulating vascular permeability, as well as angiogenesis, among other functions (30). PCs, in conjunction with other cells, participate in VC (**Figure 1**). For example, within atherosclerotic lesions, PCs can undergo osteogenic differentiation. This process can be either induced by a pro-inflammatory microenvironment, along with growth factors and cytokines secreted by ECs, or facilitated by intraplaque neovascularization, which allows PCs to be delivered into the lesion (31). PCs and vascular smooth muscle cells (VSMCs) can transform into cells resembling osteoclasts and produce calcifying matrix vesicles (32). When cultured in standard medium under different oxygen tensions, PCs increase the expression of osteopontin, osteocalcin, matrix γ-carboxyglutamic acid protein (MGP), and type I collagen, which resembles the changes observed in calcified vessels (33).

The tunica adventitia contains various cells such as macrophages, T cells, B cells, dendritic cells, and mast cells, which are present in small numbers under physiological conditions but increase significantly during inflammatory and atherosclerotic processes. Macrophages play complex roles in the processes of bone and vascular calcification. As a key component of the innate immune system, macrophages have diverse origins. During the embryonic stage, tissue-resident macrophages derived from myeloid progenitors in the yolk sac and fetal liver, such as osteoclasts and endosteal macrophages, persist into adulthood through self-renewal. These cells directly participate in the mineralization and homeostatic remodeling of the bone matrix by secreting factors like BMP2 (34, 35). In adulthood, tissue macrophages are maintained not only by local proliferation but also, to a greater extent, by monocytes derived from hematopoietic stem cells in the bone marrow. These monocytes enter the circulatory system in response to inflammatory or injury signals and migrate to pathological sites, such as the arterial wall, where they further differentiate into macrophages (36). In the pathological context of vascular calcification, macrophages from different origins can participate in inflammatory responses and the calcification process, with their polarization states and functions being finely regulated by multiple factors.

Vascular calcification is not an isolated behavior of a single cell type; rather, it is co-regulated by the constituent cells of blood vessels through a complex communication network. Endothelial cells modulate the phenotypic switching of VSMCs by releasing EVs, gaseous signaling molecules, and small-molecule peptides, thereby directly promoting the initiation and progression of vascular calcification (37). Mesenchymal stem cells play a dual role in vascular calcification: they can either promote calcification through osteogenic differentiation or exert anti-calcific protective effects via paracrine signaling, such as by blocking BMP2 signaling or downregulating Wnt signaling (38). Pericytes act as “reserve osteoblasts” in vascular calcification. In response to pathological signals, they can transdifferentiate from vascular wall guardians into osteoblast-like cells, directly contributing to calcification (31). This review focuses on macrophages, which are central cells of the innate immune system. Their high plasticity enables them to exert dual roles in vascular calcification by polarizing into different phenotypes.

### 2.2 Classification of vascular calcification

In the human body, calcium is primarily stored in bones and teeth. The calcification in bones and teeth is a physiological process, but calcium deposits can also abnormally appear in other areas, such as blood vessels, cartilage, heart valves, etc., a condition known as ectopic calcification. Among them, VC can be divided into intimal calcification, medial calcification, and heart valve calcification based on the anatomical location and clinical manifestations.

Intimal calcification typically occurs in the large arteries, particularly the coronary arteries, and is linked to hypercholesterolemia. Hence, it is also known as atherosclerotic Intimal Calcification (**Table 1**), which refers to a mineralization phenomenon occurring in the intima layer of the blood vessel near lipid or cholesterol deposits within the atherosclerotic plaque. From a histological perspective, it is characterized by eccentric deformation of the lumen and thickening of the intima layer, which gradually extends into the media layer. From a radiological perspective, it shows irregular, striped, or patchy calcification in the arteries. This type of VC can lead to clinical complications such as ischemia and arterial infarction, which are caused by atherosclerosis. The main inducing factors for intimal calcification include hypertension, diabetes, obesity, and dyslipidemia. The main pathophysiological mechanisms include inflammation, oxidative stress, and apoptosis of ECs and lipoprotein molecules (39, 40).

**Table 1:** Classification and characteristics of vascular calcification.

Feature	Intimal calcification	Medial calcification	Heart valve calcification
Anatomical Location	Vascular intima (within atherosclerotic plaques)	Tunica media of the artery	Aortic valve, mitral valve, etc.
Primary Associated Diseases	Atherosclerosis, Hypercholesterolemia	CKD, Diabetes, Aging	Hemodynamic stress, Aging, Chronic Kidney Disease
Pathological Features	Eccentric plaques, associated with lipid core	Diffuse, circumferential, independent of lipids	Leaflet thickening, deformation, reduced mobility
Cellular Drivers	Foam cells, Inflammatory cells	Osteogenic differentiation of VSMCs	Osteogenic differentiation of VICs
Imaging Presentation	Irregular, speckled, or patchy	Tram-track or tubular pattern	Thickened valves with hyperechoic nodules on echocardiography
Major Clinical Consequences	Plaque rupture, Ischemia, Myocardial Infarction	Arterial stiffness, Increased pulse pressure, Heart failure	Valve stenosis or regurgitation, Heart failure

Medial calcification is characterized by ossification of the arterial media layer in the absence of cholesterol deposits, leading to arterial stiffness (**Table 1**). Calcification of the arterial tunica media can be observed even in the smallest arteries of the human body. In this type, the calcification process resembles the formation of non-endochondral bone and teeth, where VSMCs of the media layer gradually differentiate into an osteogenic phenotype, without involving foam cells and the development of atherosclerosis. Unlike intimal calcification, medial calcification appears as tram-like or tubular, non-stenotic hyperplastic wall thickness on imaging studies. Disturbances in calcium and phosphorus metabolism, aging, inflammation, diabetes, and chronic kidney disease are recognized risk factors for this type of calcification (41).

Heart valve calcification, including the aortic and mitral valves, is caused by dystrophic calcium phosphate deposition resulting from factors such as mechanical injury, oxidative stress (OS), and inflammation (42, 43). Echocardiographic examination reveals thickening, deformation, and reduced opening amplitude of the calcified valve leaflets (44). Calcification of heart valves can lead to stenosis, subsequently triggering heart failure. Heart valve calcification is highly prevalent among hemodialysis (HD) patients, with approximately 45%-60% of patients experiencing mitral valve mineralization and 50%-60% experiencing aortic valve calcification (45). Furthermore, these heart valve calcifications exhibit significant progression within a year and contribute to increased arrhythmias, cardiovascular mortality, and morbidity in these patients.

### 2.3 Reversal of vascular calcification

VC exhibits numerous similarities to skeletal mineralization. During physiological bone remodeling, osteoblasts and osteoclasts maintain a dynamic equilibrium. Osteoblasts synthesize bone matrix proteins, which are subsequently mineralized, while coordinating with osteoclast-mediated bone resorption. When this equilibrium between osteogenesis (bone formation) and osteolysis (bone resorption) is disrupted, pathological outcomes such as VC or aberrant bone remodeling (e.g., osteoporosis) may develop (46). VC is not merely a passive mineral deposition but an actively regulated process resembling bone formation, involving osteogenic-like differentiation of VSMCs, ECM remodeling, and inflammatory regulation (47, 48). This characteristic provides a biological basis for its reversibility.

The reversal of VC relies on the coordinated regulation of multiple pathways, including inhibition of osteogenic differentiation pathways (e.g., BMP2/Smad (49), Wnt/β-catenin (50), IL-6/STAT3 (51)), promotion of autophagy (e.g., AMPK/mTOR (52), Nrf2-ARE (53), SIRT1/CXCR4 (54)), and modulation of inflammation and oxidative stress (e.g., NF-κB (55), TGF-β/Smad (56)). Targeted interventions for these pathways, such as small molecule inhibitors, gene editing, or natural compounds, may offer novel strategies for clinical treatment. Gallic acid has been demonstrated to alleviate calcification by modulating the BMP2-Smad1/5/8 pathway (57). Shenyuan granules inhibit the Wnt/β-catenin pathway by regulating serum soluble Klotho (sKlotho) levels, thereby reducing VC in chronic kidney disease (CKD) models (58). In TCF21 knockout mice, vitamin D3 and nicotine-induced VC was significantly attenuated, suggesting that targeting the IL-6/STAT3 pathway may hold therapeutic potential (51). Melatonin enhances autophagy via the AMPK/mTOR/ULK1 pathway, mitigating calcification in VSMCs (52). Luteolin suppresses CXCR4 expression by activating SIRT1, promoting autophagy, and reducing calcification (54). Sulfur dioxide (SO_2_) alleviates VC by downregulating the TGF-β/Smad pathway (56).

## 3 The fundamental biology of macrophages

Macrophages are diverse immune cells consisting of various subpopulations that possess the ability to adapt efficiently to the microenvironment through changes in their phenotype and physiological attributes. When activated, macrophages generate diverse growth-promoting factors, proteolytic enzymes, and inflammatory mediators, thereby exerting a vital function in inflammation, host defense mechanisms, and maintaining tissue balance (5). This is a group of immune cells characterized by high plasticity and functional heterogeneity. Based on their activation status and functional characteristics, they are primarily classified into two polarization states: the classically activated pro-inflammatory type and the alternatively activated anti-inflammatory type. The latter (commonly termed M2) can be further subdivided into four functional subtypes: M2a, M2b, M2c, and M2d.

**Figure 2:**
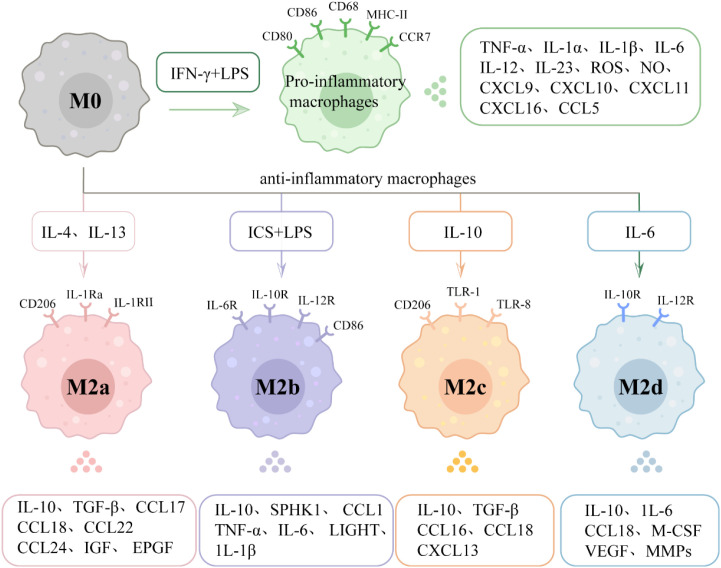
Polarization of macrophages. Macrophages primarily exhibit two polarization forms, namely pro-inflammatory and anti-inflammatory types, which can be polarized into these two categories under different cellular stimulation conditions. The latter (often referred to as M2) can be further divided into four subtypes: M2a, M2b, M2c, and M2d. Different macrophages possess distinct surface markers, which can be used to differentiate between them. During disease progression, the two types of macrophages secrete different cytokines, thereby exerting distinct effects.

Pro-inflammatory macrophages (**Figure 2**) are induced by Interferon-gamma (IFN-γ) combined with lipopolysaccharide (LPS) or bacterial products (e.g., Toll-like receptor (TLR) ligands). Their surface markers include CD80, CD86, CD68, MHC-II, and CCR7 (59). These cells predominantly secrete pro-inflammatory cytokines (TNF-α, IL-1β, IL-6, IL-12, IL-23), reactive oxygen species (ROS), and nitric oxide (NO) (60). Through the release of these inflammatory mediators and bioactive molecules, Pro-inflammatory macrophages directly eliminate pathogens (61)and recruit immune cells such as neutrophils to infection sites to amplify the inflammatory response (62). However, it is important to note that their overactivation may cause tissue damage and is closely associated with pathological processes like chronic inflammation and sepsis (63).

M2a macrophages (**Figure 2**) are induced to differentiate by IL-4 and IL-13. In humans, they exhibit high expression of the macrophage mannose receptor (CD206), interleukin-1 receptor antagonist (IL-1Ra), and Interleukin-1 Receptor type II (IL-1RII), while in mice, their characteristic markers include arginase-1 (Arg-1), FIZZ1, and Ym1/2 (64, 65). These cells secrete anti-inflammatory molecules such as IL-10, TGF-β, and CCL17/18/22/24 (66, 67). It is noteworthy that Arg-1 is widely recognized as a classic marker for murine M2a macrophages, but it is typically not expressed or is expressed at notably low levels in human anti-inflammatory macrophages, reflecting a pivotal difference in macrophage polarization mechanisms across species (68, 69). This subtype participates in tissue repair and fibrosis by promoting collagen deposition and fibroblast activation (65, 70). It also demonstrates anti-parasitic infection capabilities (71), but may play a dual role in allergic diseases (65). M2b macrophages (**Figure 2**) are activated by immune complexes, TLR ligands, or IL-1β. Their surface markers include IL-6R, IL-10R, IL-12R, and CD86 (64). These cells secrete molecules such as TNF-α, IL-1β, IL-6, and CCL1, which exhibit both pro-inflammatory and anti-inflammatory properties. They can activate the Th2 immune response and modulate immune balance, playing a critical role in chronic inflammation and autoimmune diseases. In the tumor microenvironment, they may promote immune escape (72, 73). M2c macrophages (**Figure 2**) are induced by IL-10, glucocorticoids, or apoptotic cell signals (e.g., phosphatidylserine). In humans, they express CD206, TLR-1, and TLR-8, while in mice they are characterized by Arg-1 expression. These cells secrete IL-10, TGF-β, chemokines (CCL16/18, CXCL13) (62), as well as MMPs and their tissue inhibitors (TIMPs) (70). Their key functions include clearing apoptotic cells, promoting inflammation resolution, and maintaining tissue homeostasis through suppression of excessive immune responses (64, 66, 70). M2d macrophages (**Figure 2**) are activated by adenosine, IL-6, or TLR ligands under hypoxic conditions or within tumor microenvironments. They are characterized by secreting vascular endothelial growth factor (VEGF), IL-10, and matrix remodeling enzymes (74). This subtype supports wound healing through pro-angiogenic and tissue remodeling functions, but often exhibits pro-tumor phenotypes (e.g., tumor-associated macrophages, TAMs) in tumor microenvironments (75).

While the traditional pro-inflammatory/anti-inflammatory dichotomy holds reference value (6), it oversimplifies the complex states of macrophages *in vivo*. In pathological microenvironments, macrophage phenotypes present as a continuous spectrum and should be more accurately described based on their specific stimulation conditions, gene expression profiles, and functions (76) (such as “IFN-γ/LPS-activated” or “IL-4-polarized”). Technologies such as single-cell RNA sequencing (scRNA-seq) have greatly expanded our understanding of macrophage heterogeneity, revealing new subpopulations like TREM2hi, proliferative, and interferon-inducible cells (IFNICs), which are difficult to classify using the pro-inflammatory/anti-inflammatory framework. These subpopulations play unique roles in atherosclerosis and vascular calcification (77).

The characteristics of these functionally heterogeneous subpopulations are closely related to their cellular origins and the microenvironment they reside in. Macrophages primarily originate from embryonically seeded, self-sustaining tissue-resident populations and populations continuously recruited and derived from circulating monocytes during adulthood (78). During the process of vascular calcification, macrophages of these diverse origins and activation states collectively form a complex network that regulates the osteogenic transdifferentiation of vascular smooth muscle cells and calcium deposition. This is achieved by sensing signals such as high phosphate levels and oxidative stress, secreting cytokines or vesicles, and modulating local metabolism (e.g., ATP/PPi). Understanding this network is key to elucidating disease mechanisms and developing targeted therapies. The specific mechanisms underlying these roles will be discussed in detail below.

## 4 The role of macrophages in vascular calcification

### 4.1 IFN-γ and LPS activated macrophages (pro-inflammatory macrophages) promote vascular calcification

#### 4.1.1 Pro-inflammatory macrophages secrete pro-inflammatory factors to induce calcification

**Figure 3:**
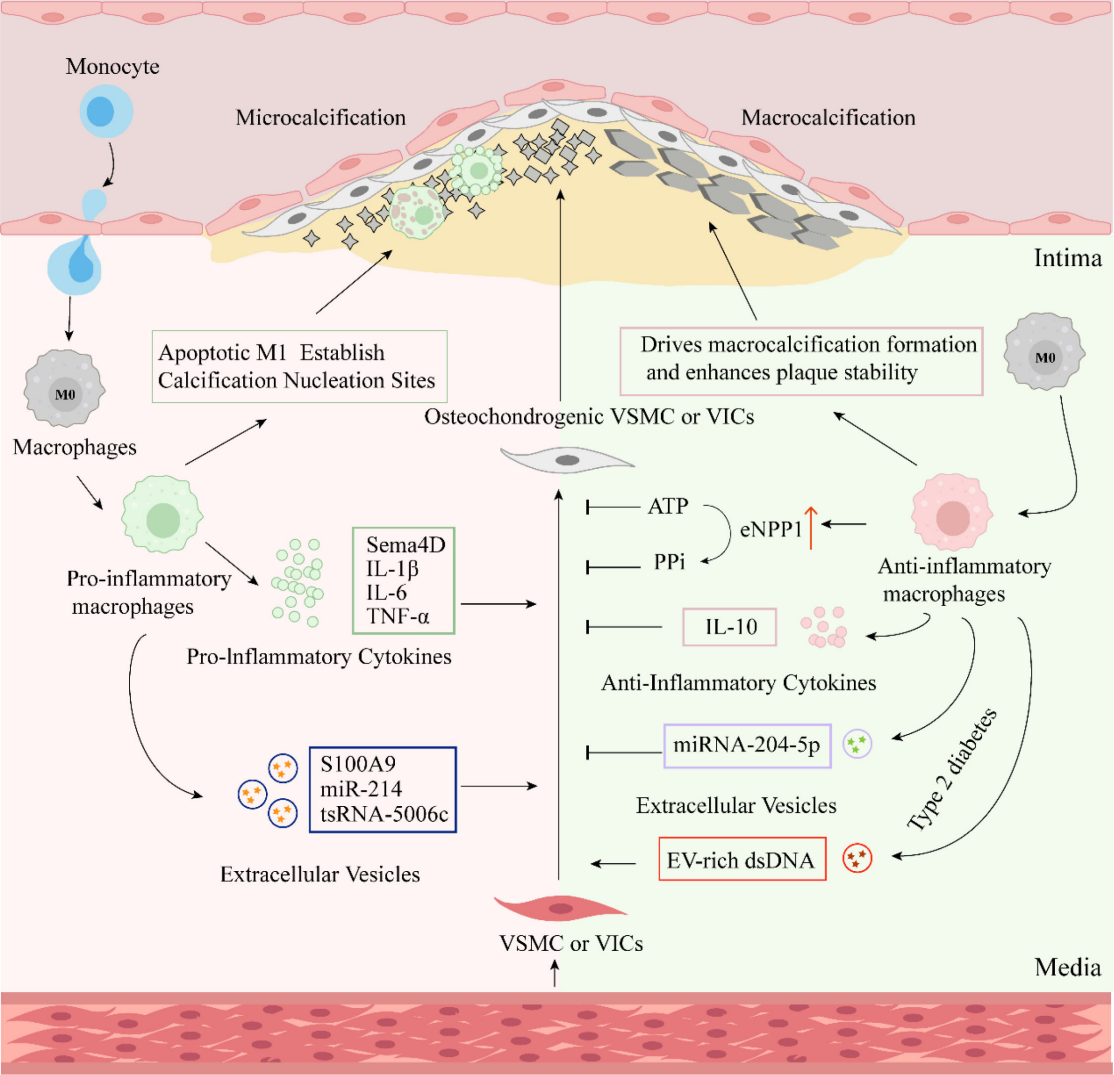
Schematic diagram of the role of macrophages in vascular calcification. Pro-inflammatory macrophages promote vascular calcification by secreting pro-inflammatory factors and EVs. Anti-inflammatory macrophages inhibit vascular calcification and enhance plaque stability through the ATP/PPi axis, as well as the release of anti-inflammatory factors and exosomes. However, under different pathological conditions or microenvironments, Anti-inflammatory macrophages may also play a promotive role in vascular calcification.

Pro-inflammatory macrophages constitute a core inflammatory mechanism driving VC through the secretion of multiple pro-inflammatory factors (**Figure 3**). First, Pro-inflammatory macrophages co-stimulated with LPS and IFN-γ highly express and secrete the axon guidance molecule Sema4D. This factor acts directly on VSMCs, leading to increased alkaline phosphatase activity, enhanced calcium deposition, and upregulation of osteogenic markers (such as RUNX2), thereby promoting calcification; notably, neutralizing Sema4D significantly inhibits this calcification process, demonstrating its critical role (79). Secondly, under inflammatory stimulation (such as priming by lipopolysaccharide LPS and activation by cholesterol crystals), the Rac1 signaling axis in macrophages is activated, thereby driving the secretion of IL-1β. This increased IL-1β then induces vascular smooth muscle cells to initiate an osteogenic transcriptional program, ultimately leading to the deposition of calcium salts within atherosclerotic plaques (8).This mechanism is clinically relevant, as serum IL-1β levels are significantly elevated in patients with high coronary artery calcification (80). Additionally, IFN-γ and LPS activated macrophages contribute to atherosclerotic calcification via TNF-α-mediated upregulation of carbonic anhydrase I (CA I)and CAII expression in VSMCs (81). Furthermore, TNF-α activates osteogenic differentiation signaling in VSMCs through upregulation of key regulators including Runx2, BMP-2, and alkaline phosphatase (ALP), thereby promoting their phenotypic transition towards osteoblast-like cells (81). TNF-α antibody can block the promotion of calcification in smooth muscle cells mediated by infection-associated macrophages (82).

#### 4.1.2 Apoptotic pro-inflammatory macrophages establish calcification nucleation sites

During vascular intimal calcification, apoptotic bodies and necrotic debris released by dying Pro-inflammatory macrophages—enriched with phospholipids and calcium-binding proteins—serve as initial nucleation sites for calcium phosphate crystals (**Figure 3**). These structures accelerate hydroxyapatite crystallization via electrostatic adsorption of calcium ions (83). If apoptotic macrophages are not promptly cleared, they will accumulate and expand the necrotic core. Within this core, microcalcifications (<15 μm) serve as nucleation sites for calcification and can coalesce into larger calcified areas. The microenvironment of the late-stage necrotic core—characterized by factors such as inflammation and matrix alterations—further promotes the progression of calcification. (84).In atherosclerotic plaques, microcalcifications are observed adjacent to apoptotic macrophages, and the degree of calcification is positively correlated with the apoptotic index of macrophages within the plaque (85). Molecularly, endoplasmic reticulum stress induces macrophage apoptosis via the TRPC3 channel (deficiency of TRPC3 can significantly inhibit late-stage calcification) (86), while the CML/RAGE axis activates the BMP-2-Cbfα1-ALP calcification cascade by initiating apoptosis (87). It is noteworthy that Pro-inflammatory macrophages promote early microcalcification (which increases plaque rupture risk) through apoptosis, while sustained inflammation may facilitate the fusion of microcalcifications into macrocalcifications (potentially stabilizing plaques) (87).

#### 4.1.3 Macrophage-derived extracellular vesicles: molecular delivery vehicles driving vascular calcification

EVs are a class of membranous vesicles released by cells (such as macrophages) into the extracellular environment, including exosomes and microvesicles, among others. They play a significant role in the process of VC (**Figure 3**). Calcification Initiation and Nucleation: The accumulation and aggregation of EVs can trigger the nucleation process of hydroxyapatite crystals (88). More importantly, EVs (particularly matrix vesicles (MVs) released by macrophages) directly initiate calcification by interacting with matrix proteins to induce Ca²^+^ influx into the vesicles (89). For instance, within atherosclerotic plaques, MVs released by macrophages drive vascular microcalcification by forming phospholipid complexes via S100A9 and Annexin A5 (Anx5) protein, thereby promoting hydroxyapatite crystallization (85).

Regulation of Calcification via Cargo Delivery: LPS-induced macrophages secrete EVs (such as microvesicles, MVs) that carry specific RNA molecules and factors to promote calcification by regulating the biological behavior of target cells (such as VSMCs and valvular interstitial cells, VICs) through paracrine actions. Pro-inflammatory macrophages deliver miR-214 to VICs via secreted MVs, downregulating the expression of its target gene TWIST1, thereby promoting aortic valve calcification (AVC) (9). IFN-γ and LPS activated macrophages secrete EVs containing tsRNA-5006c, regulating the osteogenic differentiation and calcification of aortic valvular interstitial cells from the perspective of mitophagy (90). miR-32 within pro-inflammatory macrophage-derived EVs accelerates arterial calcification in type 2 diabetic mice by suppressing autophagy in VSMCs (91). LPS-activated macrophages exacerbate VC by secreting EVs enriched with pro-inflammatory factors and specific proteins, which induce inflammation, oxidative stress, and osteogenic transformation in VSMCs (92).

Accelerating Role in Pathological Environments: Under specific pathological conditions, such as a diabetic hyperglycemic environment, the S100A9-RAGE signaling axis is activated, prompting macrophages to release calcification factor-enriched EVs. This further accelerates the formation of microcalcification within atherosclerotic plaques, thereby increasing cardiovascular risk (93). In summary, EVs derived from Pro-inflammatory macrophages play a critical promotive role in vascular and valvular calcification through multiple mechanisms: initiating calcium salt deposition, delivering regulatory molecules (such as miRNAs, tsRNAs, and pro-inflammatory factors), and being abnormally activated in pathological environments.

### 4.2 The dual role of IL-4 polarized macrophages (anti-inflammatory macrophages) in vascular calcification

Anti-inflammatory macrophages are a polarized phenotype of macrophages, primarily characterized by their anti-inflammatory and tissue-repair functions. During VC, their roles mainly include participating in inflammation regulation, promoting tissue repair, and influencing calcification stability; however, their effects may exhibit dual roles under varying pathological conditions.

#### 4.2.1 Anti-inflammatory macrophages release anti-inflammatory factors and exosomes to inhibit vascular calcification

IL-4 polarized macrophages release anti-inflammatory factors, and their derived exosomes inhibit VC (**Figure 3**). In the calcium oxalate nephrocalcinosis model, activation of the aryl hydrocarbon receptor (AhR) with FICZ treatment promotes anti-inflammatory polarization of macrophages, leading to significantly elevated levels of anti-inflammatory factors such as IL-10 secreted by these cells. This alleviates calcification by suppressing PRO-INFLAMMATORY macrophage-driven inflammatory responses and reducing calcium salt deposition (94). CD163^+^ macrophages are activated by hemoglobin-haptoglobin (HH) complexes derived from intraplaque hemorrhage. They inhibit atherosclerotic vascular calcification by activating the NF-κB pathway in VSMCs, which promotes hyaluronan synthase expression and hyaluronic acid production (10). Furthermore, C21 treatment stimulates macrophages to release exosomes carrying miRNA−204−5p, which attenuate phosphate-induced vascular calcification as modeled in hyperphosphatemia of CKD. This occurs through targeting and inhibiting RUNX2 mRNA, thereby blocking the Wnt/β-catenin signaling pathway and reducing the expression of calcification-related proteins (such as BMP-2 and OCN) (11).

#### 4.2.2 Role of anti-inflammatory macrophages in plaque stabilization

Atherosclerotic plaque rupture, triggered by structural instability features such as thinning of the fibrous cap and enlargement of the necrotic core, leads to acute thrombus formation and ultimately precipitates fatal vascular events including myocardial infarction and stroke (95). Consequently, enhancing plaque stability through therapeutic interventions constitutes an essential clinical strategy. The exosomes derived from IL-4 polarized macrophages (Exo^M2^) inhibit the proliferation, migration, and phenotypic switching of VSMCs from a contractile to a synthetic state, thereby attenuating atherosclerosis (AS) progression and enhancing plaque stability (96). In advanced plaques, macrophages within macrocalcified areas exhibit a reparative phenotype, demonstrating capabilities to modulate the ECM and express osteoclast genes. This facilitates the displacement of inflammatory foci (e.g., the lipid necrotic core), thereby promoting plaque stability (97). During plaque regression, Anti-inflammatory macrophages promote the resolution of inflammation by secreting anti-inflammatory factors such as IL-10, while simultaneously driving the formation of macrocalcification and enhancing plaque stability (98) (**Figure 3**).

#### 4.2.3 Anti-inflammatory macrophages inhibit ectopic calcification via the ATP/PPi axis

Anti-inflammatory macrophages exert anti-calcifying functions by synergistically elevating the levels of extracellular ATP and its hydrolysis product pyrophosphate (PPi) (**Figure 3**). This is achieved through enhancing β-oxidation-dependent ATP synthesis and upregulating the activity of ectonucleotide pyrophosphatase/phosphodiesterase 1 (eNPP1) (12). The mechanism by which ATP and PPi inhibit calcification lies primarily in their ability to directly bind to the surface of calcium phosphate crystals, physically hindering crystal growth and deposition (99). Furthermore, high phosphate concentrations induce the conversion of unpolarized macrophages into a novel type of phosphate-activated macrophage (MPiφs). These cells counteract ectopic calcification induced by hyperphosphatemia by increasing the release of extracellular ATP and pyrophosphate (PPi), thereby inhibiting mineral deposition. MPiφs macrophages are phenotypically closer to the broad anti-inflammatory class of macrophages but possess unique characteristics, and are thus defined as a novel macrophage subtype (100).

#### 4.2.4 Distinct roles of anti-inflammatory macrophages in vascular calcification across specific pathological contexts

Under various pathological conditions and/or their corresponding microenvironments, Anti-inflammatory macrophages promote VC (**Figure 3**). Bacteroides fragilis-derived extracellular vesicles carrying double-stranded DNA activate macrophages, leading to the up-regulation of the pro-calcification factor Serpine1 and thereby exacerbating vascular calcification in type 2 diabetes (T2D). This represents a previously unrecognized mechanism of gut microbiota-immune interaction in diabetic vascular complications (13). Furthermore, under pathological microenvironments characterized by high Intercellular Adhesion Molecule-1 (ICAM-1) and low Inorganic Pyrophosphate (PPi), Anti-inflammatory macrophages may fail to effectively execute their expected tissue-repair and anti-calcification functions, or their phenotype/function may become dysregulated, thereby failing to prevent—and potentially even indirectly promoting—the calcification process (101).

## 5 Macrophage-based diagnostic strategy for vascular calcification

### 5.1 Discovery and application of macrophage-associated biomarkers

In research on biomarkers for VC, multiple molecules secreted by macrophages hold significant clinical value due to their temporal expression patterns during calcification progression, unique detection advantages, and specific applicability to patient populations (**Table 2**). miR-32 serves as an early-response marker, upregulated early in type 2 diabetes (T2D) patients under hyperglycemic conditions. Its plasma-based non-invasive detection, combined with good pathological specificity and clinical utility, makes it suitable for the early monitoring of T2D-related VC (T2D VC) (91). For the diagnosis of T2D VC, serum Serpine1 (secreted by Anti-inflammatory macrophages) levels are significantly elevated (1.68-fold) and demonstrate high diagnostic value (13). Sema4D is a marker applicable to multiple high-risk populations (such as heart failure, chronic kidney disease (CKD), atherosclerosis, and diabetes). Its advantages lie in its mechanism-driven nature (directly promoting osteogenic differentiation of VSMCs, circulatory detectability (easily quantifiable soluble sSema4D), and potential therapeutic relevance (enabling integrated targeted therapy and efficacy monitoring) (79). Regarding the assessment of calcification activity in specific populations, macrophage highly expressed CA II, leveraging its easily detectable enzymatic activity, is suitable for evaluating pathological calcification activity levels in CKD patient cohorts (102). Concerning late-stage calcification progression and cardiovascular risk assessment, macrophage-secreted OPN, specifically expressed within late-stage calcified plaques, is particularly applicable for evaluating calcification progression and associated cardiovascular risk in end-stage renal disease (ESRD) patients following peritoneal dialysis (PD) or hemodialysis (HD) (102).

**Table 2:** Summary and outlook of diagnostic and therapeutic strategies targeting macrophages in vascular calcification.

Strategy category	Specific cases/methods	Main targets/mechanisms	Current challenges/future directions
Diagnostic & Monitoring Strategies	Biomarkers: miR-32, Sema4D (sSema4D), Serpine1, CA II, OPN	Early response (miR-32), high diagnostic value for T2D-related VC (Serpine1), clear mechanism and detectable in circulation (Sema4D), assessment of calcification activity (CA II), evaluation of late-stage calcification progression (OPN).	Combining imaging techniques with traditional calcification imaging for more comprehensive biological status assessment. Achieving real-time, high-resolution *in vivo* monitoring.
	Imaging Techniques: Optical Coherence Tomography (OCT), CD206-targeted PET, anti-MARCO MRI/Upconversion Nanoparticles (UCNPs)	Shows macrophage infiltration co-localized with calcification foci (OCT), with specific imaging of anti-inflammatory macrophages via CD206-PET and pro-inflammatory macrophages via MARCO-MRI.	
Therapy	Polarization Modulation: Artesunate, Curcumin, Ginsenoside, Titanium Nanotubes (TNTs), Luteolin, other flavonoids	Signaling pathways (e.g., NF-κB, HIF-1α, PPARγ, AMPK, SIRT1) to inhibit pro-inflammatory or promote anti-inflammatory polarization.	*In vivo* specificity, long-term safety, development of synergistic multi-target drugs. Overcoming functional heterogeneity of Anti-inflammatory macrophages in different pathological environments.
	Inhibiting Recruitment & Activation: MIF inhibitors, Etidronate, Inorganic Pyrophosphate (PPi)	CXCR2/CXCR4 receptors, macrophage aggregation process.	Potential side effects from systemic immunosuppression; development of targeted delivery systems.
	Engineered Therapies: CRISPR/Cas9 gene-edited macrophages, Engineered exosomes (e.g., carrying miRNA-204-5p)	Introducing anti-inflammatory or pro-phagocytic genes; delivering therapeutic molecules to target cells (e.g., VSMCs) to block osteogenic signals (e.g., Wnt/β-catenin).	Manufacturing complexity, *in vivo* survival rate and colonization, immunogenicity, ethical issues.
	Modulating Microbiome-Immune Axis: Targeting gut microbiota-derived EVs (e.g., *Bacteroides fragilis*-derived EVs)	Signaling pathways in macrophages (e.g., STING-Mef2d), influencing their polarization and pro-calcific functions.	Establishing direct causality in VC, significant individual variability, indirect effects.

It should be noted that the biomarkers discussed above (e.g., miR-32, Sema4D, Serpine1, CA II, OPN) are not exclusively secreted by macrophages. Under various pathological conditions, other cell types—such as endothelial cells, vascular smooth muscle cells, fibroblasts, and immune cells—may also contribute to their production (103–106). Therefore, while these molecules are associated with macrophage activity in vascular calcification, their elevated levels in circulation or tissues may reflect a broader inflammatory or calcific microenvironment. Future studies utilizing cell-specific deletion models, single-cell transcriptomics, or spatial proteomics are warranted to precisely delineate the macrophage-specific contribution to these biomarkers in VC.

### 5.2 Applications of imaging techniques in macrophage monitoring

Imaging techniques play a pivotal role in the non-invasive assessment of VC, particularly in visualizing macrophage activity and polarization within calcified plaques. The dynamic balance between PRO-INFLAMMATORY and Anti-inflammatory macrophages influences plaque stability and calcification progression. Therefore, imaging modalities that specifically target macrophage subsets offer promising tools for early detection, risk stratification, and therapeutic monitoring in VC (**Table 2**). Clinical optical coherence tomography (OCT) evidence demonstrates that the degree of macrophage infiltration within calcified plaques—especially when macrophages are closely co-localized with calcification foci—shows a significant correlation with a series of established or newly proposed high-risk (vulnerable) plaque morphological features. These features include increased microcalcification and more severe inflammatory infiltration (107). In recent years, molecular imaging techniques targeting distinct functional macrophage subsets have provided powerful new tools. On one hand, a novel PET tracer targeting the macrophage mannose receptor CD206 has been validated in a rat experimental acute myocardial infarction (MI) model, demonstrating its ability to specifically image the infarct region, with signal intensity correlating positively with anti-inflammatory macrophage abundance (108). On the other hand, an upconversion nanoprobe targeting the MARCO receptor (anti-MARCO UCNPs) enables optical/MRI dual-modality non-invasive imaging, allowing for the specific detection of pro-inflammatory macrophage polarization within atherosclerotic vulnerable plaques (109). These two techniques, specifically targeting the pro-inflammatory and anti-inflammatory subsets respectively, open new avenues for monitoring the activity and progression risk of VC. It is hypothesized that the combined application of these techniques (calculating the pro-inflammatory/anti-inflammatory ratio, analyzing spatial distribution) and integration with traditional calcification imaging information holds promise for achieving a more comprehensive and earlier assessment of the biological state of VC (such as the balance between inflammatory activity and repair). This integrated approach could thus play a critical role in risk stratification, guiding personalized treatment, and evaluating therapeutic efficacy in clinical settings.

## 6 The therapeutic role of macrophages in the treatment of vascular calcification

### 6.1 Compounds targeting vascular calcification through regulation of macrophage pro-inflammatory/anti-inflammatory polarization

Certain compounds can inhibit the conversion of macrophages to the pro-inflammatory phenotype or promote their transformation into the anti-inflammatory phenotype, thereby playing a role in delaying VC (**Tables 2**, **3**). Artesunate, a derivative of artemisinin, inhibits the generation of pro-inflammatory macrophages by regulating the HIF-1α and NF-κB signaling pathways, thereby exerting anti-atherosclerotic effects. (110). Curcumin, a diketone compound extracted from the rhizomes of *Curcuma longa*, alleviates cadmium-induced generation of pro-inflammatory macrophages by suppressing the NF-κB and NLRP3 inflammatory signaling pathways (111). Titanium dioxide nanotubes (TNTs) promote the generation of anti-inflammatory macrophages by inhibiting glycolysis and activating the AMPK signaling pathway, which may contribute to reducing the risk of calcification in the long term (114). Ginsenoside Rb1 promotes the secretion of IL-4 and IL-13 from macrophages, thereby activating the STAT6 signaling pathway, which drives the generation of anti-inflammatory macrophages and ultimately enhances the stability of atherosclerotic plaques (112). Ginsenoside Rg3 modulates the polarization of macrophages from a pro-inflammatory towards an anti-inflammatory phenotype by targeting PPARγ and alleviates atherosclerosis, suggesting its potential therapeutic effect on VC (113). Other flavonoid compounds, including quercetin, resveratrol, kaempferol, proanthocyanidins, luteolin, and acacetin, have been shown to promote macrophage differentiation into anti-inflammatory macrophages (115, 116). Evidence also suggests their potential therapeutic role in VC (54, 117–119). However, further verification is required to determine whether these compounds delay VC through macrophage polarization modulation.

**Table 3:** Small-molecule compounds targeting macrophage polarization and their mechanisms.

Compound/intervention	Source/type	Effect on polarization	Primary mechanism of action	Experimental evidence in VC
Artesunate (110)	Artemisinin derivative	Inhibits pro-inflammatory macrophages	Regulates the HIF-1α and NF-κB signaling pathways	Attenuates atherosclerosis in models
Curcumin (111)	Curcuma longa rhizome	Inhibits PRO-INFLAMMATORY MACROPHAGES	Suppresses the NF-κB and NLRP3 inflammatory signaling pathways	Validated in a cadmium-induced atherosclerosis model
Ginsenoside Rb1 (112)	Ginseng	Promotes anti-inflammatory macrophages	Enhances IL-4/IL-13 secretion and activates STAT6 phosphorylation	Improves atherosclerotic plaque stability
Ginsenoside Rg3 (113)	Ginseng	Promotes anti-inflammatory macrophages	Targets PPARγ	Alleviates atherosclerosis in diabetic models
Titanium Nanotubes (TNT) (114)	Nanomaterial	Promotes anti-inflammatory macrophages	Activates the AMPK pathway to inhibit glycolysis	Drives anti-inflammatory polarization *in vitro*, reducing the risk of calcification
Luteolin (54)	Natural Flavonoid	Promotes anti-inflammatory macrophages	Activates SIRT1, promoting autophagy	Mitigates calcification in VC models via autophagy
Other Flavonoids (Quercetin, Resveratrol) (115, 116)	Various Plants	Promote anti-inflammatory macrophages	Modulation of various anti-inflammatory and signaling pathways	Literature suggests anti-inflammatory and immunomodulatory potential, supporting a potential role in VC
Statins (98, 120)	Statins	Promote anti-inflammatory macrophages	alleviate chronic inflammation within the plaques and promotes plaque regression	induces VSMCs to undergo orderly osteogenic differentiation and maturation
Epigallocatechin gallate (121)	Flavonoids	reducing the pro-inflammatory polarization	inhibits the TBK1/cGAS/STING/NLRP3 signaling pathway	suppressing vascular calcification and related atherosclerosis

In addition to natural compounds, some synthetic drugs have shown potential to influence vascular calcification by modulating macrophage polarization. Statins, through their anti-inflammatory effects, can promote the polarization of macrophages within atherosclerotic plaques toward the anti-inflammatory phenotype. This shift in polarization not only helps alleviate chronic inflammation within the plaques and promotes plaque regression but also induces VSMCs to undergo orderly osteogenic differentiation and maturation, thereby facilitating the formation of macrocalcification. Such structurally ordered calcified deposits can enhance the mechanical stability of plaques, ultimately stabilizing them and reducing the risk of plaque rupture (98, 120). Epigallocatechin gallate (EGCG) inhibits the TBK1/cGAS/STING/NLRP3 signaling pathway, reducing the generation of pro-inflammatory macrophages, thereby suppressing vascular calcification and related atherosclerosis (121).

### 6.2 Inhibition strategies for macrophage recruitment and activation

Macrophages are key effector cells of the innate immune system, typically derived from monocytes recruited from the bloodstream to sites of pathology, where they differentiate. Their role in the process of VC is multifaceted and complex (**Table 2**). Inhibiting the recruitment and activation of macrophages essentially represents an interventional strategy targeting the inflammatory core of VC. Macrophage migration inhibitory factor (MIF) promotes immune cell aggregation and the progression of atherosclerosis by activating C-X-C Motif Chemokine Receptor 2 (CXCR2) and CXCR4 receptors; studies indicate that blocking MIF signaling can reverse plaque progression, suggesting its potential as a therapeutic target for atherosclerosis (122). In Abcc6-deficient mice, cardiac calcification is closely associated with macrophage accumulation and the formation of multinucleated cells, and administration of etidronate or inorganic pyrophosphate (PPi) can effectively prevent calcification by inhibiting this process (101). Furthermore, strategies targeting macrophage recruitment, polarization status, regulating exosome secretion functions (11), and modulating the microbiota-immune axis (13) have all demonstrated potential in delaying the progression of VC. The current major challenges lie in translating these strategies into clinically effective therapies and overcoming issues such as cell-specific targeting and long-term safety. Future research is expected to focus more on developing targeted delivery systems (e.g., engineered exosomes), regulating gut microbiota, and identifying new diagnostic markers and therapeutic targets.

## 7 Current controversies and challenges

### 7.1 Technical bottlenecks in dynamic monitoring of macrophage polarization states

Current techniques for dynamically monitoring macrophage polarization status still face technological challenges. Although existing studies have assessed the pro-inflammatory phenotype by detecting markers such as IL-6, iNOS, and CXCL9 (123), and evaluated the anti-inflammatory phenotype using specific markers like ARG-1, YM-1, and TGF-β1 (124), these static indicators struggle to reflect the dynamic changes during the polarization process. Detection techniques for markers include flow cytometry, immunohistochemistry, Western blot, and others. Recent studies have attempted to utilize exosomes as monitoring tools to distinguish between pro-inflammatory and anti-inflammatory macrophages (11, 91), but their specificity in the VC microenvironment remains to be validated. Exosomes are typically isolated using differential ultracentrifugation combined with commercial kits for purification, and are identified through Western Blot and transmission electron microscopy (11, 91). Current technologies also lack the ability to effectively distinguish between functional anti-inflammatory macrophage subsets under steady-state conditions and those undergoing phenotypic drift driven by disease (97). Given the significant dual role of anti-inflammatory macrophages in the calcification process across different pathological microenvironments, this technological bottleneck severely impedes the precise spatiotemporal regulation and utilization of their dynamic transition from homeostatic to disease-driving phenotypes in VC treatment strategies.

In recent years, researchers have developed several novel methods for detecting macrophage polarization. For instance, the combination of atomic force microscopy (AFM) and artificial intelligence (AI) offers significant advantages in label-free, dynamic monitoring and functional correlation, making it particularly suitable for in-depth mechanistic studies and analyzing cellular states in complex microenvironments (125). Single-cell RNA sequencing enables the unbiased discovery of new macrophage subtypes and reveals their high heterogeneity and dynamic changes during pathological processes, serving as a powerful tool for deciphering complex microenvironments (126). Advanced nanoprobe strategies hold the potential for non-invasive, *in vivo*, real-time monitoring of macrophage polarization status and spatial distribution in pathological sites such as tumors or vascular calcification (127). Although these emerging technologies demonstrate great potential, their complexity, low throughput, and high cost currently limit their routine application in practical analyses. Further advancement is still required for their clinical translation.

### 7.2 Challenges in targeting anti-inflammatory polarization for vascular calcification therapy

Therapeutic strategies targeting anti-inflammatory polarization face the challenge of determining the critical time window. In atherosclerotic plaques, this specific polarization towards an anti-inflammatory phenotype is associated with features of plaque regression. However, it also influences the calcification process through various mechanisms, such as suppressing BMP-2-dependent pro-calcific signaling and impairing osteoclast-like functions (86, 128). As a result, while Anti-inflammatory macrophages contribute to overall plaque stability, they may not effectively prevent or resolve calcified deposits. Furthermore, although Anti-inflammatory macrophages generally suppress VC, they exhibit pro-calcific effects under specific pathological conditions, such as type 2 diabetes (13). However, due to the lack of systematic evaluation of anti-inflammatory macrophage functions across all disease models and pathological conditions related to VC, their precise role in other disease contexts remains unclear.

### 7.3 Species differences between human and murine macrophages and atherosclerosis models

Research on macrophage polarization and the evaluation of its role in vascular calcification must fully consider species differences. First, there are significant distinctions in cellular phenotypic markers. For example, Arg-1 is a classic marker for murine M2a macrophages but is not a reliable indicator for their human counterparts (68, 69). More importantly, in the crucial pathological context of atherosclerosis for vascular calcification, there is a fundamental divergence in the immune cell composition of plaques between humans and mice. An integrated single-cell RNA sequencing study revealed that immune cells in mouse atherosclerotic aortas are predominantly myeloid cells (including macrophage subsets), whereas T cells predominate among immune cells in human carotid plaques (129). This finding holds significant implications for vascular calcification research: although macrophages may be the central inflammatory cells driving calcification in experimental mouse models, T cell-mediated adaptive immune responses likely play a more critical role in human disease. Currently, apart from the clear marker differences for the M2a subtype, there is a lack of systematic comparative studies on the functional homology, molecular definitions, and specific roles in vascular calcification of other anti-inflammatory subsets (such as M2b, M2c, and M2d) between humans and mice. This constitutes an important uncertainty that must be cautiously addressed when translating conclusions from mouse models to human disease. Therefore, when discussing the role of macrophages in vascular calcification, especially in atherosclerosis-related calcification, it is essential to specify the species background of the research model and to interpret the translational significance from mouse models to human disease with caution.

## 8 Discussion

Given the dual role of macrophages in VC and its potential for multi-target interventional strategies, future research should focus on translating fundamental mechanistic findings into clinical applications. Artificial intelligence-assisted multi-target drug design can systematically integrate macrophage polarization-related signaling pathways (e.g., BMP/Smad, Wnt/β-catenin, IL-6/STAT3, etc.) to screen compound combinations that synergistically regulate the balance between pro-inflammatory and anti-inflammatory phenotypes. Additionally, the development of biomimetic nanocarriers (such as liposomes or engineered EVs) enables precise delivery of polarization-modulating drugs, enhancing drug concentration at the lesion site while reducing systemic side effects. Engineering macrophages—for instance, by knocking in anti-inflammatory or pro-phagocytic genes via CRISPR/Cas9 technology—provides a new direction for cell therapy, enabling them to actively recognize and repair calcified lesions, thereby achieving true precision immunotherapeutics.

Current immunometabolic regulation of VC requires breakthroughs in spatiotemporally specific technologies. Although existing studies have revealed dynamic changes in macrophage subsets across different stages of calcification, there is still a lack of *in vivo*, real-time, high-resolution monitoring and intervention methods. Future efforts should further develop molecular imaging technologies (e.g., multimodal probes designed to visualize the pro-inflammatory and anti-inflammatory phenotypes) combined with single-cell spatiotemporal transcriptomics to decipher the functional heterogeneity of macrophages within the calcification microenvironment. On this basis, developing spatiotemporally specific regulatory strategies—such as optogenetic control of local polarization—will become a priority. Gene editing (e.g., AAV-mediated delivery of polarization regulators) and metabolic interventions (e.g., modulating ATP/PPi balance) are expected to enable precise reprogramming of macrophage phenotypes at the lesion site, thereby inhibiting calcification while promoting vascular repair.

In summary, macrophages, as central cellular players linking immunity and metabolism, offer unique advantages in the treatment of vascular calcification (VC). First, their high plasticity enables phenotypic switching through external interventions, allowing for upstream control of the calcification process. Second, macrophages can simultaneously influence inflammation, mineralization, and repair via multiple mechanisms—such as the secretion of anti-inflammatory factors, extracellular vesicles (EVs), and the regulation of ATP/PPi metabolism—thereby providing potential for multifunctional synergistic therapy. Third, while macrophage-associated biomarkers combined with imaging techniques can offer real-time feedback for disease staging and efficacy evaluation, it must be noted that most of these molecules are not macrophage-specific; their expression in calcified vessels can be influenced by multiple cell types. Hence, future research should aim to identify more specific macrophage-derived signatures and employ advanced techniques such as single-cell RNA sequencing and multiplex imaging to clarify the cell-type-specific origins of biomarkers. Although challenges remain, including the dynamic monitoring of polarization states and functional heterogeneity under pathological conditions, macrophages remain an ideal target for achieving precise immunotherapy in VC. Interdisciplinary strategies will be essential to advance the full translation from mechanistic research to clinical application.
